# Prior knowledge is essential for the beneficial effect of targeted memory reactivation during sleep

**DOI:** 10.1038/srep39763

**Published:** 2017-01-04

**Authors:** Sabine Groch, Thomas Schreiner, Björn Rasch, Reto Huber, Ines Wilhelm

**Affiliations:** 1 University Children’s Hospital Zürich, Zürich, Switzerland; 2 Donders Institute for Brain, Cognition and Behaviour, Radboud University Nijmegen, The Netherlands; 3 Department of Psychology, University of Fribourg, Switzerland; 4 Department of Child and Adolescent Psychiatry and Psychotherapy, Psychiatric Hospital, University of Zürich, Switzerland; 5 Department of Psychiatry, Psychotherapy and Psychosomatics, Psychiatric Hospital, University of Zürich, Switzerland; 6 Department of Psychology, University of Zürich, Switzerland

## Abstract

Prior knowledge speeds up system consolidation and accelerates integration of newly acquired memories into existing neocortical knowledge networks. By using targeted memory reactivations, we demonstrate that prior knowledge is also essential for successful reactivation and consolidation of memories during sleep, both on the behavioral and oscillatory level (i.e., theta and fast spindle activity). Thus, prior knowledge is a prerequisite for new memories to enter processes of system consolidation during sleep.

System memory consolidation refers to a long-lasting process whereby a newly encoded labile hippocampal memory trace becomes stabilized and permanently stored in the neocortex^1^,^2^,^3^. This process can be speeded up when neocortical networks of associated knowledge exist into which the newly acquired memory trace can be incorporated^4^,^5^,^6^,^7^. It has been assumed that the overlapping reactivation of new memory traces in the hippocampus and associated neocortical networks during sleep enables the integration of new information into existing neocortical networks^8^,^9^. Thus, memory reactivations should most efficiently promote the consolidation of new memories when neocortical networks of prior knowledge are present. However, this has not been tested so far. The present study aims to start filling this gap by investigating the effects of targeted memory reactivation on the consolidation of newly acquired memories that either can or cannot be related to prior knowledge.

## Results

### The effect of memory cueing on retention performance

**Figure 1:**
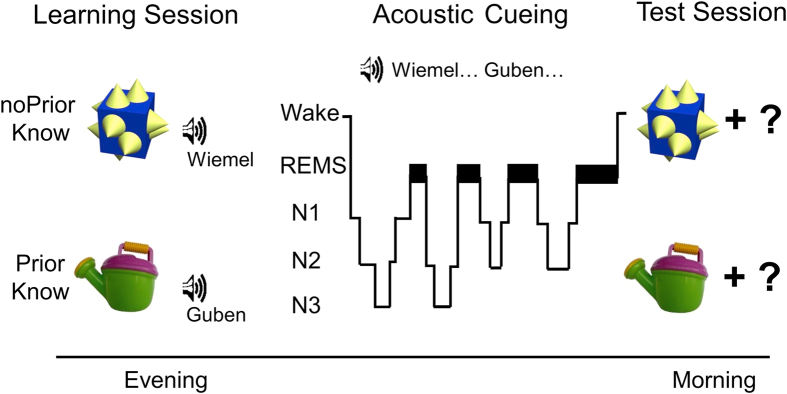
Task and design. In the learning session in the evening, participants encoded associations of acoustically presented pseudo-words and visually presented familiar objects (stimuli related to prior knowledge, “PriorKnow”) and novel objects (stimuli not related to prior knowledge, “noPriorKnow”). Half of the words from both conditions were presented again during post-learning NonREM sleep (stage N2 and N3) in order to induce the reactivation of the associated information. A typical polysomnogram visualizes the proportion of sleep stages during the nocturnal retention interval (wake (W), non-rapid eye movement (NonREM) sleep stages 1–3 (N1–N3), REMS). In the Test Session the next morning, memory recall was tested for all picture-word associations in a cued recall procedure.

**Figure 2:**
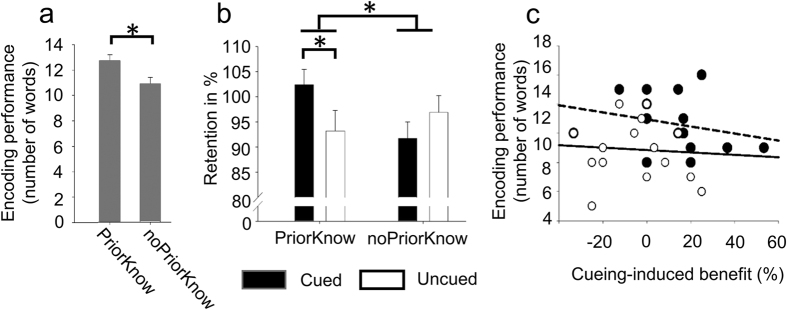
Memory performance. (**a**) In the learning session, participants encoded a higher number of information related to prior knowledge (“PriorKnow”) than information not related to prior knowledge (“noPriorKnow”). (**b**) Cueing benefited memory retention the next morning only for stimuli related to prior knowledge but not for stimuli not related to prior knowledge (interaction between “cueing” x “condition” and post-hoc t-test are indicated). The relative difference between recall and learning performance with learning performance set to 100% is indicated. (**c**) Encoding performance was not correlated with cueing-induced benefit neither for stimuli related (filled circles) nor stimuli not related to prior knowledge (open circles). *P ≤ 0.05; Mean ± SEM are indicated.

Participants learnt associations between pseudo-words (e.g. “Wiemel”) and pictures showing familiar objects (e.g. a watering can; i.e. stimuli that are related to prior knowledge, “PriorKnow”) or novel objects (stimuli that are not related to prior knowledge, ”noPriorKnow”; see Fig. 1 for a description of the procedure and of the task). Half of the words from both conditions were presented again via loudspeakers during subsequent NonREM (Rapid Eye Movement) sleep. This method also referred to as “memory cueing” is known to induce the reactivation of associated memories^10^,^11^,^12^. In the morning after sleep, memory was tested for all picture-word associations. In the evening before sleep, participants showed higher learning performance for stimuli related to prior knowledge as compared to stimuli not related to prior knowledge (t(15) = 2.86; *P *= 0.012; Fig. 2a). Cueing during sleep improved later recall with this critically depending on the existence of prior knowledge. More specifically, memory recall was higher for cued as compared to uncued stimuli when prior knowledge was available (t(15) = 2.14, *P *= 0.05; Fig. 2b). However, no such improvement was present without prior knowledge (*P *> 0.41; interaction ‘cueing’ x ‘condition’: F(1,15) = 5.21; *P *= 0.037). Importantly, performance at encoding was not associated with the beneficial effect of memory cueing neither for stimuli related to prior knowledge (r = −0.26, *P *> 0.33) nor for stimuli not related to prior knowledge (r = −0.07, *P *> 0.79; Fig. 2c).

### Oscillatory correlates of memory cueing during sleep

**Figure 3:**
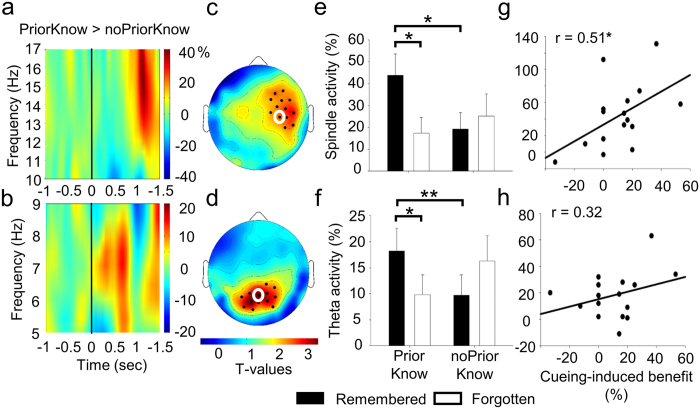
EEG activity in response to memory cueing. (**a,b**) Time-frequency plots indicate the difference in the subsequent memory effect (later remembered minus later forgotten stimuli) for stimuli related to prior knowledge (“priorKnow”) and stimuli not related to prior knowledge (“noPriorKnow”) in the theta frequency range (a, time-frequency data at electrode number 62 is indicated, for a position of this electrode on the scalp see white circle in c) and the fast spindle frequency range (b, time-frequency data at electrode number 104 is indicated, for a position of this electrode on the scalp see white circle in d). Stimuli related to prior knowledge showed a higher response than stimuli not related to prior knowledge in the theta (5–8 Hz) and in the fast spindle frequency range (14–16 Hz). (**c,d**) The topographical distribution of the difference in the SME is shown for mean activity in the theta band for a time interval between 500–800 ms after cue onset (**d**) and in the fast spindle band for a time interval between 1100–1300 ms (**c**). T-values are indicated. The difference in the SME was most pronounced for spindle activity in a right fronto-temporal cluster (*P* = 0.039, corrected for multiple comparisons) and for theta activity in a parietal cluster (*P* = 0.035, corrected for multiple comparisons). Significant electrodes (*P* < 0.05) are indicated as black dots. (**e,f**) Mean fast spindle and theta activity in response to later remembered and later forgotten cues is indicated for stimuli related and stimuli not related to prior knowledge. Later remembered as compared to later forgotten cues induced higher fast spindle (**e**) and theta activity (**f**) only for stimuli related to prior knowledge. Mean ± SEM are indicated. (**g,h**) Scatterplots indicate the correlation between fast spindle (**g**) and theta response (**h**) to remembered cues and the cueing-induced benefit (memory recall for cued minus uncued stimuli) for stimuli related to prior knowledge. Fast spindle activity but not theta activity was significantly correlated with the cueing-induced benefit. *P < 0.05; **P < 0.01.

In a next step, we aimed to uncover the underlying neurophysiological correlates of the beneficial effect of cueing for stimuli related to prior knowledge but not for stimuli not related to prior knowledge. To analyze the induced oscillatory responses to cues during post-learning periods of sleep, we calculated the subsequent memory effect (SME,^13^), i.e. the difference in the induced oscillatory response to cues associated with later remembered and cues associated with later forgotten stimuli in both conditions. Based on previous findings demonstrating a role of theta and fast spindle activity in the successful reactivation of memories^14^,^15^, statistical analyses were restricted to these frequency bands. The SME differed between stimuli related to prior knowledge and stimuli not related to prior knowledge in the fast spindle frequency range (14–16 Hz; Fig. 3a) and in the theta frequency range (5–8 Hz; Fig. 3b). More specifically, the SME did significantly differ between both conditions in an electrode cluster over the right fronto-temporal cortex in the fast spindle frequency band, 1100–1300 ms after cue onset (*P* = 0.039, corrected for multiple comparisons; Fig. 3c) and in a cluster over the centro-parietal cortex in the theta frequency band, 500–800 ms after cue onset (*P* = 0.035, corrected for multiple comparisons; Fig. 3d). Later remembered as compared to later forgotten induced higher theta as well as fast spindle activity for stimuli related to prior knowledge (theta: t(15) = 2.48, *P* = 0.026; spindle: t(15) = 2.55, *P* = 0.022; Fig. 3e,f) but not for stimuli not related to prior knowledge (theta: t(15) = −1.82, *P* > 0.09; spindle: t(15) = −0.69, *P* = 0.50). Moreover, later remembered information related to prior knowledge induced a higher response than later remembered information not related to prior knowledge in both frequency bands (theta: t(15) = 2.22, *P* = 0.043; spindle: t(15) = 3.23, *P* = 0.006). Finally, fast spindle but not theta activity in response to later remembered information related to prior knowledge was correlated with the beneficial effect of cueing (spindle: r = 0.51, *P* = 0.042, theta: r = 0.32, *P* = 0.22; Fig. 3g,h).

## Discussion

Here, we show for the first time that the existence of prior knowledge is an essential prerequisite for the beneficial effect of cueing on memory consolidation during sleep. More specifically, we found that cueing of newly acquired information benefits their later recall only if the cued memories can be related to prior knowledge. Our findings on increased theta and fast spindle activity in response to memory cues related to prior knowledge are in line with recent findings demonstrating that both oscillatory events are related to the successful reactivation of memories^14^,^15^. Importantly, they go beyond these earlier findings in showing that the increase in theta and spindle activity critically depends on the existence of prior knowledge. The temporal and topographical distribution of both oscillatory events indicates their unique role in the successful reactivation of memories. The earlier theta response to word cues over the parietal cortex might reflect the immediate reinstatement of the memory trace in the brain region of initial storage^16^,^17^. The later spindle response over frontotemporal regions which predicted the cueing-induced memory benefit might reflect the successful integration of new information into these neocortical networks and their stabilization^17^,^18^,^19^,^20^. Future studies need to experimentally manipulate theta and spindle activity in response to memory cues with different level of prior knowledge in order to empirically test the exact role of these oscillations in the reinstatement and the integration of these memories into the existing memory networks. In sum, our findings provide first empirical evidence for recent theoretical assumptions^1^,^8^,^9^ indicating that overlapping reactivations of newly acquired hippocampal memory traces together with associated neocortical networks of prior knowledge facilitate the integration of new information into these memory networks in the process of memory consolidation.

Participants remembered a higher number of words associated with a familiar object than words associated with a novel object. This is in line with a number of previous studies demonstrating that the existence of prior knowledge benefits the encoding of new memories^21^,^22^,^23^,^24^. Can these differences at encoding account for the effect of memory cueing merely for information that can be related to prior knowledge? First studies indeed point towards the notion that factors acting during encoding essentially modulate the effect of memory cueing. More specifically, performance level at encoding was found to be positively associated with the beneficial effect of memory cueing^14^,^25^. However, in our study the correlation between performance level at encoding and the cueing-induced benefit was not significant. Nevertheless to finally exclude any impact of differences at encoding, future studies should 1) experimentally manipulate or at least control factors acting at the time of encoding that come along with prior knowledge such as the depth of encoding and 2) experimentally induce familiarity within an experiment by using the same kind of novel stimuli.

Since we did not include a wake control group, i.e. a group in which memory cueing took place during wakefulness one could argue that the cueing effects are not limited to the sleep period. A number of previous studies showed no or even detrimental effects of cueing on memory consolidation including various kinds of wake retention intervals such as cueing during daytime wakefulness and cueing during night-time active or passive waking^10^,^14^,^26^,^27^. The reactivation of memories during sleep and wakefulness is assumed to result in the destabilization of these memories^26^,^28^. Due to the presence of slow oscillations, sleep spindles, a low cholingeric tone and the lack of interference these labile memory traces are again strengthened during sleep^3^. During wakefulness, all these factors are absent which on the one hand can explain the vulnerability of these memories to interference and on the other hand can explain why memory cueing during wakefulness is not effective. Thus, it appears to be very unlikely that cueing during sleep and wakefulness lead to comparable effects on the consolidation of newly acquired memories with and without prior knowledge.

## Methods

### Participants

Sixteen adults between 18–25 yrs (*M* *=* 20.25, *SEM* = 1.95 yrs; 11 females) participated in the study. Participants were recruited via advertisements placed at the university, at the children’s hospital and in local newspapers. Interviews as well as standardized questionnaires ensured that the participants had no behavioural problems, cognitive impairments or sleep disorders. More specifically, we assessed in a telephone screening interview (for recruitment) and in a more elaborate interview (at the beginning of the adaption night) the information of life-time and current irregularities in sleeping behaviour, learning behaviour and general diseases. All participants were asked for individual sleep habits, i.e., usual time to go to bed, time getting up, etc. in order to schedule both nights in the sleep laboratory in accordance with their usual sleep habits. Also, participants took notes on their daily activities, sleep time and time to get up in a sleep diary for the seven days prior to the experiment which was additionally compared to and supplemented by actigraphical monitoring. Participants did not take any medication at the time of the experiment, and the ingestion of caffeine or alcohol was not allowed on experimental days. The study was approved by the local ethics committee (Kantonale Ethikkommission Zürich) and procedures were carried out in accordance with the approved guidelines. Participants gave written informed consent before participating.

### Design and Procedure

Participants were adapted to polysomnographic recordings on a night preceding the experiment proper. Experimental and adaptation night in the sleep lab were separated by at least one night at home to exclude possible effects of bad sleep quality in the adaptation night on sleep in the experimental night. In the experimental night, participants came to the sleep lab around 2.5 hrs before participants’ habitual bedtime. First, the electrode net was placed. Immediately thereafter, participants performed the learning task (including learning and immediate recall of picture-word associations) and went to bed afterwards. During NonREM sleep, half of the learnt words were presented via loudspeaker placed behind the participants’ head to stimulate the reactivation of the respective picture-word associations. The next morning, participants were awakened at their habitual wake time. Memory retention was tested ~45 min later to avoid any modulating effect of sleep inertia on recall performance (see Fig.1 for the experimental procedure).

### Associative memory task

The associative memory task included forty pairs of objects (20 familiar objects and 20 novel objects) and pseudo-words (see Fig. 1 for example of pairs). All pseudo-words were disyllabic, phonotactically legal in German, were stressed on the first syllable as common in German, had a consonant–vowel onset, and had typical masculine or neuter endings. They were spoken slowly by a young woman with a mean duration of 809 ms per word, are digitized at a rate of 44.1 kHz, and were presented through a loudspeaker with an intensity of approximately 65 dB sound pressure level during learning. In the learning session, participants were instructed to memorize each picture-word pair for recall immediately after learning and for later recall the next morning. All picture-word pairs were presented three times in a randomized order. In the first two presentation trials (without feedback) every picture was shown on the screen for a total of 4000 ms. The associated word was additionally presented acoustically via loudspeakers 1500 ms after picture onset. Before presenting each picture, a fixation cross was shown on the screen for 800–1200 ms. In the third learning trial, all pictures were presented again on the screen for 2500 ms, followed by a question mark indicating that the participants had to recall the associated word. Thereafter, the correct word was presented again and participants had the possibility to memorize the picture-word pair for another 5500 ms before the next picture was presented. This last learning trial was followed by a cued recall test which was comparable to the last learning trial except for the fact that no feedback was given. In case of not reaching the criterion of 16 correct responses in this cued recall test, two additional learning trials without feedback and another cued recall test were administered. Memory retention was again tested in the morning in the same cued recall procedure. In all recall tests, participants were required to speak out loud the remembered word to the experimenter who stood next to them and who recorded memory performance. All words that were identical with the learnt word or included the word-stem of the learnt word were counted as correct. A second experimenter double-checked after the task was completed whether the first experimenter had correctly judged the accuracy of each word recall. As a measure of memory consolidation, we calculated the percentage of performance at recall with performance during learning set to 100% (110% means that a participant remembered 10% more words at recall than during learning).

### Memory cueing during sleep

During post-learning periods of NonREM sleep (sleep stages N2 and N3), words that had been paired with a picture during the learning phase were presented again via loudspeaker (with a 55 dB sound pressure level). Half of the learnt words was cued, whereas the other half of the learnt words was not cued. Half of the cued words were randomly and individually chosen from the group of words that had been correctly remembered (“hits”) by an individual during the learning phase, whereas the other half was taken from the group of forgotten words (“misses”). For example, given that a participant correctly remembered twenty of the forty picture-word associations - half of them from each of the two categories - this would result in cueing of five correctly remembered stimuli related to prior knowledge, five forgotten stimuli related to prior knowledge, five correctly remembered stimuli not related to prior knowledge and five forgotten stimuli not related to prior knowledge. Words were presented with an inter-stimulus-interval of 6 s with a random jitter of 0 to 0.4 s at a maximum of 22 times for each word (mean number of cues related to prior knowledge: 204.69 ± 11.07, cues not related to prior knowledge: 205.31 ± 11.08; *P* > 0.56). Note that including the number of cue presentations as a covariate does not change the results (interaction ‘cueing’ x ‘condition’: *P* = 0.047). A jitter was included in order to ensure that cues reach the slow waves at variable points. Slow waves have been found to be causally related to memory consolidation during sleep^29^,^30^. Whether slow waves are also implicated in the stabilization of reactivated memories and whether presenting the cues at a specific phase of the slow wave (either up- or down state) is relevant in this regard and has previously been discussed^17^ but was not empirically tested so far. On this background, it appeared to be most reasonable to increase the variability of the temporal relationship between slow waves and auditory cues by including a jitter. An experienced experimenter inspected the EEG in real-time to determine sleep stages and to detect any indicator of an arousal. Word cueing was started when a participant had spent more than 10 min in NonREM stage N3, and it was immediately stopped whenever any sign of an arousal, waking up or any other change in sleep stages was observed by the experimenter. The acoustic presentation of word cues did not disturb nocturnal sleep (see Table 1 for descriptives on sleep parameters).

### Sleep EEG

Sleep in the experimental and the adaptation night was recorded using high-density sleep EEG (Electrical Geodesics Sensor Net for long-term monitoring, 128 channels, referenced to a vertex electrode) including electromyographic and electrooculographic recordings. Data were sampled at 500 Hz (0.01–200 Hz). For sleep scoring, the EEG was bandpass filtered (0.5–50 Hz) and downsampled to 128 Hz. The EEG was visually scored by two trained individuals for sleep stages Wake, N1, N2, N3 and REM sleep at frontal, central and occipital electrodes (20 s epochs) based on American Academy of Sleep Medicine standard criteria^31^.

### Analyses of power changes in response to cues

EEG signals were preprocessed using Brain Vision Analyzer 2.0 (Brain Products, Gilching, Germany). Preprocessing included re-referencing of the raw EEG data to the average of the two mastoids and low-pass (30 Hz, roll-off 24 dB per octave) and high-pass filtering (0.1 Hz, roll-off 12 dB per octave). EEG data were epoched into segments of 1.9 s before to 2.9 s after word onset. For each individual, segments were categorized in stimuli that were and stimuli that were not remembered in the test session in the morning (i.e. referred to as “later remembered” and “later forgotten” stimuli). Succeeding EEG oscillatory analyses were performed using the open source Fieldtrip toolbox^32^ running on Matlab R2014a (MathWorks, Natick, MA). Time-frequency analysis was computed for each trial by using a 7-cycle Morlet wavelet decomposition, ranging from 2 to 30 Hz in 0.5 Hz steps. A sliding window with a step size of 10 ms was applied across the entire length of the epochs. Single trials were normalized with respect to a pre-stimulus time window ranging from −1.000 ms to −100 ms.

### Statistical analyses

Statistical analysis of memory retention was based on 2 × 2 analyses of variance (ANOVA) including the repeated measures factors ‘cueing’ (cued, uncued) and ‘condition’ (stimuli related to prior knowledge “PriorKnow”, stimuli not related to prior knowledge “noPriorKnow”). Post-hoc comparisons as well as all statistical comparisons with only one level were performed using student *t*-tests. Correlation analyses were conducted using Pearson’s correlation coefficient. The level of significance was set to P ≤ 0.05. Statistical analyses of the EEG data were performed with a nonparametric randomization test using cluster correction^33^ as implemented in FieldTrip. The cluster alpha was set to 0.05 and 500 randomizations were conducted for all tests. Clusters were considered significant at P < 0.05 (two-sided).

## Additional Information

**How to cite this article**: Groch, S. *et al*. Prior knowledge is essential for the beneficial effect of targeted memory reactivation during sleep. *Sci. Rep.* **7**, 39763; doi: 10.1038/srep39763 (2017).

**Publisher's note:** Springer Nature remains neutral with regard to jurisdictional claims in published maps and institutional affiliations.

## Figures and Tables

**Table 1:** Sleep parameters.

	Absolute time (in min)	Percentage of time (in %)
	mean ± SEM	mean ± SEM
Wake	26.94 ± 5.91	6.04 ± 1.29
Non REMS N1	37.50 ± 4.94	8.41 ± 1.09
Non REMS N2	232.69 ± 11.82	52.60 ± 2.58
Non REMS N3	58.06 ± 7.05	13.10 ± 1.56
REMS	87.50 ± 6.25	19.86 ± 1.50
TST	442.69 ± 5.72	

Sleep parameters for all participants are given in mean ± SEM of absolute time in minutes and percentage of total sleep time; REMS = rapid eye movement sleep, TST = total sleep time.
